# Sulfur and Peroxide Cross-Linking of Lignosulfonate-Filled Compounds Based on Acrylonitrile–Butadiene Rubber and Styrene–Butadiene Rubber

**DOI:** 10.3390/polym17070950

**Published:** 2025-03-31

**Authors:** Ján Kruželák, Michaela Džuganová, Andrea Kvasničáková, Ján Hronkovič, Jozef Preťo, Ivan Chodák, Ivan Hudec

**Affiliations:** 1 Department of Plastics, Rubber and Fibres, Faculty of Chemical and Food Technology, Slovak University of Technology in Bratislava, Radlinského 9, 812 37 Bratislava, Slovakia; 2 VIPO a.s., Gen. Svobodu 1069/4, 958 01 Partizánske, Slovakia; 3 Slovak Academy of Sciences, Polymer Institute, Dúbravská Cesta 9, 845 41 Bratislava, Slovakia

**Keywords:** rubber, biopolymer, sulfur curing system, peroxide curing system, cross-linking, properties

## Abstract

Calcium lignosulfonate was incorporated into rubber compounds based on styrene–butadiene rubber (SBR) and acrylonitrile–butadiene rubber (NBR) in amounts ranging from 10 to 60 phr. A sulfur-based curing system and a peroxide curing system consisting of dicumyl peroxide in combination with methacrylic acid zinc salt were used for cross-linking of the compounds. The aim of the work was to investigate the influence of lignosulfonate and curing system composition of the cross-linking process, morphology, physical–mechanical and dynamic–mechanical characteristics of the composites. The achieved results showed that peroxide cured composites demonstrated higher cross-link density, which was found not to be influenced by the content of lignosulfonate. The cross-link density of sulfur-cured composites was lower and showed a decreasing tendency with increasing amounts of the biopolymer. A lower cross-linking degree was reflected in a higher elongation at break and higher increase in the elongation at break of the corresponding composites. On the other hand, peroxide-cured composites exhibited a higher modulus M100 and higher hardness. The microscopic analysis revealed that co-agent in peroxide vulcanization contributed to the improvement of adhesion between the biopolymer and the rubber resulting in higher tensile strength of the equivalent composites. The higher cross-link density of peroxide-cured composites caused higher restriction of the chain segments’ mobility, due to which these composites exhibited a higher glass transition temperature.

## 1. Introduction

The depletion of sources, global warning, and negative effects on the environment have shifted the awareness towards bio-economy and green technologies. Lignocellulosic raw materials represent suitable alternatives to petroleum-based products, as they are highly available and sustainable and have a positive effect on global greenhouse gas reduction. Lignin is the second most widespread biopolymeric material in the world and thus bears a huge application potential. The annual production of technical lignin is about 50–70 million tons, of which only 1–2% is used to produce the value-added goods. The rest is landfilled or burned to generate energy or to recover chemicals [1,2]. Lignin exhibits highly branched amorphous aromatic structure with diversity of functional groups as carboxyls, carbonyls, hydroxyls, or methoxy groups. Due to these groups, lignin exhibits attractive properties including antimicrobial, antioxidant, UV absorption, adhesive properties, hydrophobicity, etc. [3,4,5,6,7].

There are several types of lignin depending on the processing method and extraction procedures [7,8,9,10,11]. Sulfur and sulfur-free extraction processes are the most common procedures to obtain lignin from lignocellulosic parts of wood and plants. Sulfur-based lignins include Kraft lignin and lignosulfonates. Kraft lignin is derived by Kraft pulping using sodium sulfide and sodium hydroxide at pH between 13 and 14. The sulfite pulping process is acid-catalyzed and basic (pH between 1 and 2) using an aqueous sulfur dioxide and a sodium, calcium, ammonium, or magnesium salt-based acid. When compared to Kraft lignin, lignosulfonates contain less free carboxyl and phenolic hydroxyl groups and higher amount of sulfur, mainly in the form of sulfonate groups [12,13]. Due to the presence of sulfonate groups, they are water-soluble. Lignosulfonates have been widely used as dispersant agents, surfactants, binders, compatibilizers, additives to concretes, and composites [14,15,16,17]. In addition, due to their aromatic structure, high amount of carbon, good mechanical stability, and viscoelastic properties, they are suitable materials as additives and fillers in rubber-based compounds and composites [18,19,20,21,22]. To fabricate polymer composite materials with good applicable and utility properties, good adhesion and interactions between the filler and the rubber matrix on their interfacial region must be formed. Lignins and lignosulfonates are polar materials and thus their compatibility with non-polar matrices is usually poor, often resulting in weak physical–mechanical properties of the final composites. On the other hand, polar functional moieties provide space for surface modification and thus increasing interactions with rubber matrices. Good adhesion and formation of interactions between the filler and the rubber on the filler–rubber interface is the basic principle for rubber composites’ reinforcement.

The final utility properties of rubber goods are achieved during the vulcanization or curing. Vulcanization is a physical–chemical process during which a plastic rubber compound is transformed into cross-linked highly elastic products—vulcanized by chemical reactions between functional groups of rubber and curing additives. The cross-linked network improves the dimensional stability of rubber materials; increases elasticity, mechanical, and dynamical properties; and reduces hysteresis or plasticity. A variety of curing systems have been applied for the cross-linking of rubber compounds, such as sulfur-based systems, organic peroxides, phenol–formaldehyde resins, quinones, metal oxides, amines, ureas, etc. Among them, sulfur curing systems are the most commonly used, accounting for about 90% or rubber-based articles, followed by organic peroxides.

Sulfur vulcanization is the oldest method used for cross-linking unsaturated rubbers. Sulfur is always used in combination with activators and accelerators. Sometimes, retarders of vulcanization or prevulcanization inhibitors can be part of sulfur curing systems, too. The mechanism and chemistry of sulfur vulcanization is very complex and still not comprehensibly elucidated. During the process, rubber chains segments are linked with sulfur-based cross-links having different lengths and amounts of sulfur in sulfur bridges (monosulfidic, disufidic, and polysulfidic cross-links) [23,24]. In general, sulfur-cured vulcanizates demonstrate good tensile behavior, good elastic and dynamic properties, good abrasion resistance, or good resistance to dynamic fatigue. On the other hand, due to the low bonding energy of sulfidic cross-links, their thermo-oxidative stability is usually weak [25,26,27].

Organic peroxides can be applied to vulcanize both saturated and unsaturated rubbers. By the application of organic peroxides, carbon–carbon bonds are formed between the chains [28]. C-C bonds have higher dissociation energy when compared to sulfidic cross-links, and thus the main feature of peroxide-cured rubbers is higher thermal resistance and higher resistance to degradation factors, like oxygen and heat [29,30]. On the other side, when compared to sulfur-cured vulcanizates, they usually have worse tear and tensile strength and weaker dynamic properties.

To boost the cross-linking process with organic peroxides, double bonds containing organic compounds, termed as co-agents, are often used [31,32]. The co-agents help to increase the efficiency of peroxide curing process by enhancing the cross-link density and by modifying the cross-links’ structure. Couple of reaction mechanisms have been proposed for particular types of co-agents. In general, network enhancement through the homopolymerization and grafting of co-agents between the chain segments are the main co-agents’ reaction mechanisms [33,34,35,36]. As a lot of co-agents are polar materials, they also increase the polarity of rubber compounds and enhance adhesion towards polar fillers and additives in the rubber compounding.

In this study, calcium lignosulfonate in various amounts was incorporated into NBR- and SBR-based formulations. A sulfur curing system and organic peroxide in combination with a co-agent were applied for cross-linking. The main aim was to compare the effect of vulcanization systems on the cross-linking process, physical–mechanical, and dynamic–mechanical properties of the composites.

## 2. Experimental

### 2.1. Materials

Styrene–butadiene rubber (SBR, Kralex 1502, styrene content—23.5 wt.%, Mooney viscosity ML 1 + 4 (100 °C)—52) prepared by cold emulsion polymerization was supplied from Synthos Kralupy, a.s., Kralupy nad Vltavou, Czech Republic. Acrylonitrile–butadiene rubber (NBR, SKN 3345, acrylonitrile content—31–35%, Mooney viscosity ML 1 + 4 (100 °C)—45) was supplied from Sibur International, Moscow, Russia. Calcium lignosulfonate having trade name Borrement CA120 was provided by Borregaard Deutschland GmbH, Karlsruhe, Germany. The molecular weight of lignosulfonate was 24,000 g·mol^−1^ and the specific surface area was 3.9 m^2^·g^−1^. In addition to carbon (46.63 wt.%), the biopolymer contained hydrogen (5.35 wt.%), nitrogen (0.14 wt.%), sulfur (5.62 wt.%), and hydroxyl groups (1.56 wt.%). Lignosulfonate was applied into rubber formulations in a concentration scale ranging from 10 to 60 phr. For the cross-linking of rubber compounds, sulfur and peroxide curing systems were used. The sulfur-based curing system consisted of zinc oxide (3 phr) and stearic acid (2 phr) as activators, accelerator *N*-cyclohexyl-2-benzothiazole sulfenamide CBS (1.5 phr), and sulfur as curing agent (1.5 phr). The additives of the sulfur curing system were supplied from Vegum a.s., Dolné Vestenice, Slovak Republic. The peroxide curing system consisted of dicumyl peroxide DCP as curing agent (1 phr) and methacrylic acid zinc salt ZDMA (10 phr) as co-agent. DCP and ZDMA were supplied from Sigma-Aldrich, St. Louis, MO, USA.

### 2.2. Methods

#### 2.2.1. Fabrication and Curing

The fabrication of the composites was performed in a laboratory kneader Brabender (Brabender GmbH & Co. KG, Duisburg, Germany) at 90 °C and 55 rpm. The mixing process was carried out in two steps.

First, rubber was plasticated for 1 min, then the activators of the sulfur curing system were added. Calcium lignosulfonate was introduced after 2 min, and the rubber mixture was compounded for the next 4 min. After the first step of mixing, the rubber compounds were additionally homogenized and sheeted by using a two-roll mill. Sulfur and the accelerator were introduced in the second step, and the mixing process continued in the kneader for 4 min at 90 °C with rotor speed set up to 55 rpm. Finally, the rubber compounds were sheeted in a two-roll mill.

The compounding procedure of rubber formulations with the peroxide curing system proceeded following the same conditions (90 °C, 55 rpm, overall time of mixing 10 min). The rubber and the filler were compounded in the first step, which took 6 min, and additives of curing systems were applied in the second step. Final homogenization and sheeting were accomplished in a two-roll mill.

The curing process was carried out using a Fontijne hydraulic press (Fontijne, Vlaardingen, The Netherlands) following the optimum cure time of each rubber compound. The temperature of curing was 170 °C and the press was 15 MPa. After curing, thin sheets with dimensions of 15 × 15 cm and a thickness of 2 mm were obtained.

#### 2.2.2. Determination of Curing Characteristics

An MDR 2000 oscillatory rheometer (Alpha Technologies, Akron, OH, USA) was used to determine curing isotherms and curing characteristics.

The investigated curing parameters were as follows:

*M_L_* (dN·m)—minimum torque;

*M_H_* (dN·m)—maximum torque;

∆*M* (dN·m)—torque difference, ∆*M* = *M_H_* – *M_L_*;

*t_c_*_90_ (min)—optimum curing time;

*t_s_*_1_ (min)—scorch time.

#### 2.2.3. Determination of Cross-Link Density

To calculate the cross-link density *ν*, dried and weighted samples were immersed in xylene, in which they swelled. The samples were taken out from the solvent every hour, wiped out of solvent, and weighted. When the weight of the samples was constant, equilibrium swelling was reached and used for the determination of cross-link density by the Krause-modified Flory–Rehner equation [37].

#### 2.2.4. Investigation of Physical–Mechanical Characteristics

The tensile tests were performed according to valid technical standards using Zwick Roell/Z 2.5 tearing equipment (Zwick GmbH & Co. KG, Ulm, Germany). The cross-head speed was set up to 500 mm·min^−1^ with a gauge length of 25 mm. Dumbbell-shaped specimens (width 6.4 mm, length 80 mm) were cut with a special knife from a 2 mm thick cured rubber plate. The hardness in Shore A was measured by using a durometer.

#### 2.2.5. Microscopic Analysis

The surface morphology was evaluated by a JEOL JSM-7500F scanning electron microscope (Jeol Ltd., Tokyo, Japan). The samples were first cooled down in liquid nitrogen under glass transition temperature and then fractured into small fragments with surface area of 3 × 2 mm. The fractured surface was covered with a thin layer of gold and put into the microscope. The source of electrons was a cold cathode UHV field emission gun, the accelerate voltage ranged from 0.1 kV to 30 kV, and the resolution was 1.0 nm at 15 kV and 1.4 nm at 1 kV. SEM images were captured by a CCD-Camera EDS (Oxford INCA X-ACT, Jeol Ltd., Tokyo, Japan).

## 3. Results and Discussion

### 3.1. Curing Process and Cross-Link Density

**Figure 1:**
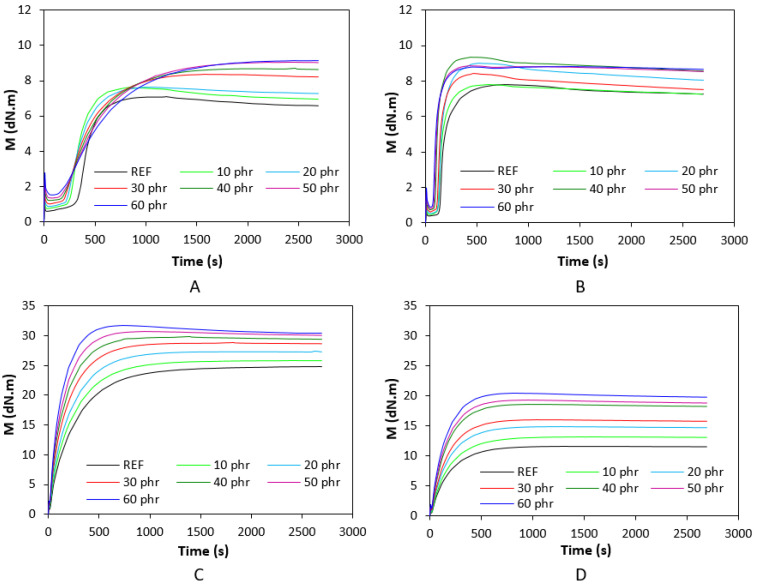
Curing isotherms of SBR-based composites cured with sulfur system (**A**). NBR-based composites cured with sulfur system (**B**). SBR-based composites cured with peroxide system (**C**). NBR-based composites cured with peroxide system (**D**).

**Figure 2:**
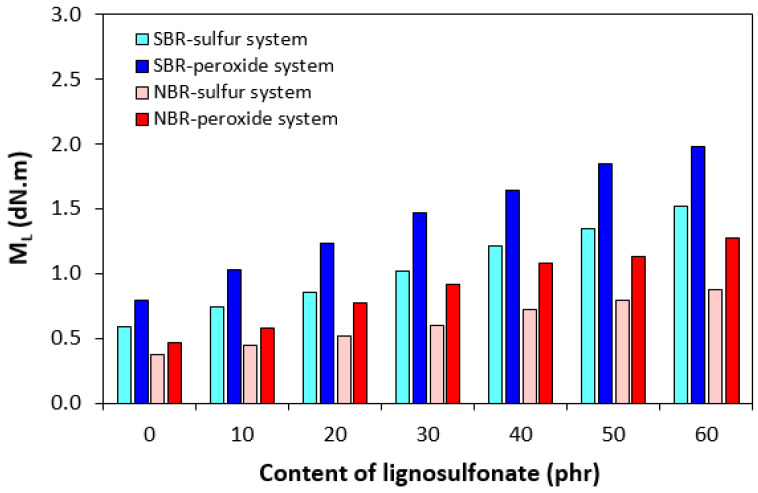
Influence of lignosulfonate content and curing system composition on minimum torque *M_L_* of composites.

The corresponding vulcanization isotherms of rubber compounds cured with the sulfur and peroxide systems are illustrated in Figure 1. It becomes apparent from them that the course of curing isotherms was influenced by the type of rubber as well as by the curing system composition. From Figure 2, it becomes apparent that the minimum torque *M_L_* of both SBR- as well as NBR-based compounds show an increasing trend with increasing content of the biopolymer. The minimum torque, to a certain extent, relates to the viscosity of the compounds before the curing process started, which suggests that the incorporation of the biopolymer resulted in an increase in viscosity. The highest minimum torque was exhibited by rubber compounds based on SBR cured with the peroxide system, followed by the equivalent compounds cured with the sulfur system. The compounds based on NBR cured with both vulcanization systems demonstrated a lower *M_L_*. This points to the higher viscosity of SBR-based rubber formulations, as SBR exhibited higher Mooney viscosity. When comparing curing systems, higher minimum torque and thus higher viscosity were manifested by rubber compounds with the peroxide system applied. The maximum torque showed an increasing trend with increasing amounts of the biopolymer, too. As shown in Figure 1A,B, the *M_H_* of the compounds based on SBR and NBR cured with the sulfur system was very similar. When comparing both types of rubber formulations cured with the peroxide system (Figure 1C,D), one can see that higher maximum torque was exhibited by those based on SBR.

**Figure 3:**
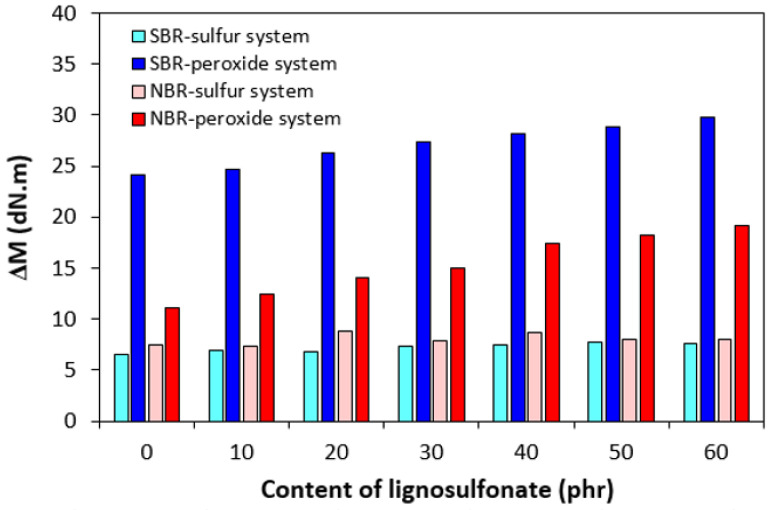
Influence of lignosulfonate content and curing system composition on torque difference Δ*M* of composites.

**Figure 4:**
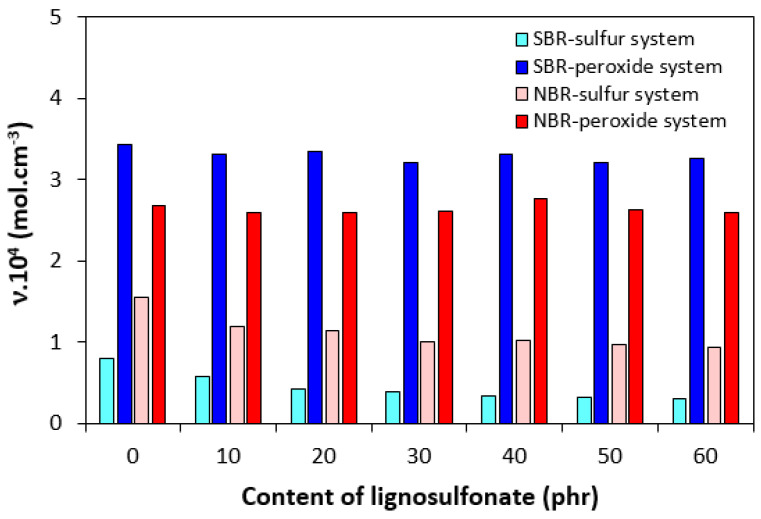
Influence of lignosulfonate content and curing system composition on cross-link density *υ* of composites.

**Figure 5:**
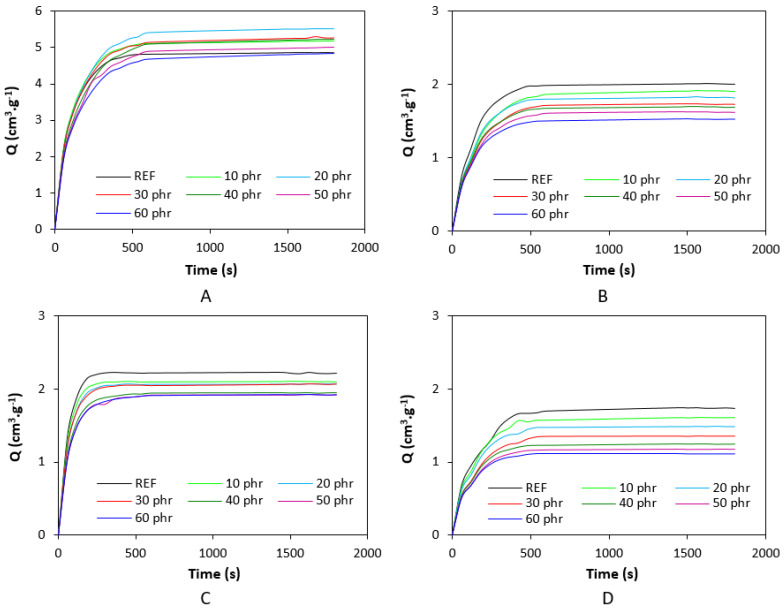
Swelling indexes of SBR-based composites cured with sulfur system (**A**). NBR-based composites cured with sulfur system (**B**). SBR-based composites cured with peroxide system (**C**). NBR-based composites cured with peroxide system (**D**).

The highest difference between the maximum and minimum torque Δ*M* was demonstrated by SBR-based formulations cured with peroxide system, followed by the equivalent compounds based on NBR (Figure 3). The SBR- and NBR-based compounds cured with sulfur system exhibited lower Δ*M*. The difference between maximum and minimum torque Δ*M* is to a large extent proportional to the cross-link density, and when comparing Figure 3 and Figure 4, one can see a very close correlation between both characteristics. The highest cross-link density was exhibited by peroxide-cured composites based on SBR having the highest Δ*M*. On the other hand, SBR-based composites cured with the sulfur system with the lowest torque difference demonstrated the lowest degree of cross-linking (Figure 4). The incorporation of lignosulfonate resulted in a slight decrease in cross-link density for both SBR- and NBR-based compounds cured with the sulfur system. On the other hand, almost no influence of the biopolymer on cross-link density was recorded for rubber formulations cured with the peroxide system. The corresponding swelling indexes are presented in Figure 5. The higher the degree of cross-linking, the shorter the chain segments between the cross-links and the lower the free volume in the matrix. With a lower free volume, a lower amount of solvent can diffuse into the compounds. As seen in Figure 5, the highest amount of xylene was diffused into the samples in the initial stages of swelling, up to 5–6 h. Equilibrium swelling was achieved after 24 h.

**Figure 6:**
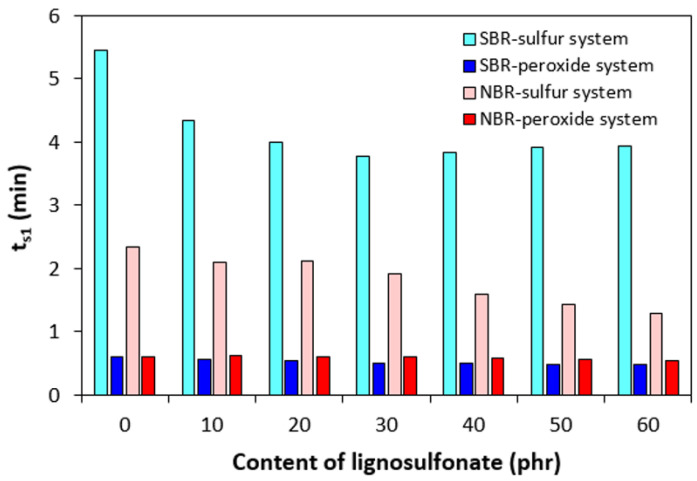
Influence of lignosulfonate content and curing system composition on scorch time *t_s_*_1_ of composites.

**Figure 7:**
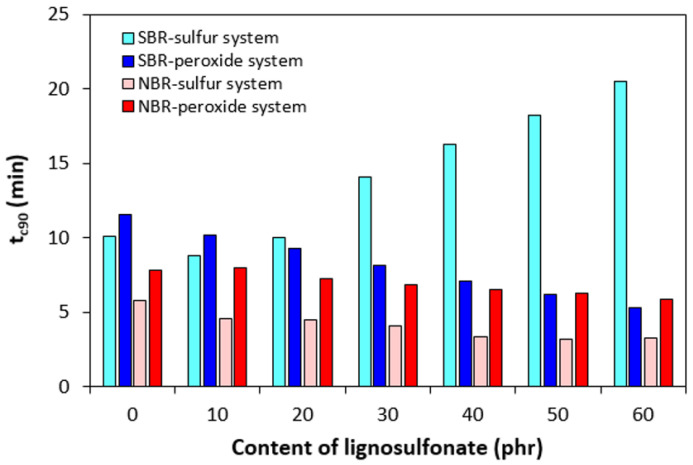
Influence of lignosulfonate content and curing system composition on optimum cure time *t_c_*_90_ of composites.

The application of lignosulfonate caused a decrease in scorch time *t_s_*_1_ for rubber formulations cured with the sulfur system (Figure 6). The scorch time of the compounds cured with the peroxide system was much lower and was found to be independent on the content of the biopolymer. With the exception of the compounds based on SBR cured with the sulfur system, the optimum cure time *t_c_*_90_ showed a decreasing trend with increasing content of lignosulfonate (Figure 7). On the other hand, a significant prolongation of the optimum cure time was recorded for sulfur-cured SBR-based formulations with high biopolymer content.

Peroxide vulcanization is a radical process, during which organic peroxide first undergoes homolytic cleavage via breaking labile oxygen–oxygen bonds at a curing temperature [38]. The formed radical species promptly react with rubber chain segments to form cross-links between them. To that corresponds a very low scorch time (Figure 6). The regulation of scorch time during peroxide vulcanization is very complicated as it is achieved only by the type of peroxide and its dissociation rate at a curing temperature [39,40,41]. It becomes apparent from Figure 6 that scorch time for both types of rubber formulations cured with the peroxide system was very similar and fluctuated by only around half a minute.

Both NBR- and SBR-based compounds cured with the peroxide system exhibited very similar curing kinetics, i.e., scorch time and optimum cure time, but differed in torque difference and cross-link density. Thus, the insight into the mechanism of vulcanization should be outlined. SBR and NBR are copolymers of styrene or acrylonitrile, respectively. Styrene and acrylonitrile structural units do not provide active sites for cross-linking. Cross-linking of both rubbers is performed exclusively via butadiene structural units [27,36,42,43,44]. The active radical species formed from peroxide decomposition can react with rubber chains in two ways. The first mechanism involves the abstraction of reactive allylic hydrogens from rubber chains to form macromolecular radicals. During the second mechanism, the peroxide-derived radicals attack the double bonds in the rubber structure, again followed by the formation of macromolecular radicals. The formed macroradicals can either mutually recombine or participate in addition reactions with the free double bonds in rubber chains, mainly those situated in 1,2-butadiene structural units. Pendant vinyl units are very prone to radical addiction mechanisms. Moreover, they are less sterically hindered and thus more accessible for radical species when compared to double bonds in the main chains (cis/trans) [45,46,47]. The chain character of addition reactions results in high cross-linking effectiveness. The highest torque difference and cross-link density were exhibited by peroxide-cured composites based on SBR. The peroxide cross-linking efficiency of NBR is lower, as NBR contains lower amount of butadiene structural units. Also, electron-withdrawing acrylonitrile groups make double bonds in NBR less reactive, so addition reactions have been reported to be of lower importance than in the case of SBR [36,48]. Thus, the equivalent NBR-based composites exhibited lower torque difference and lower cross-link density. The co-agent used in combination with dicumyl peroxide contributed to the increase in the cross-link density of both composite types, too. The specific reaction mechanism of methacrylic acid zinc salt in peroxide vulcanization is discussed in following section.

On the other hand, sulfur vulcanization of rubber compounds is a very complex process. Although it is still not clearly understood, it has been proposed that it proceeds in three general stages. In the first stage, the accelerator reacts with the activators to form a transition complex, which then reacts with sulfur to generate an active sulfurating agent. This stage is called the induction period, and the length of induction period is dependent on the composition of the sulfur curing system, mainly on the type of accelerator. The used accelerator CBS is the class of delayed-action accelerators, which is characterized by a prolonged induction period [49,50]. To that corresponds the longer scorch time of sulfur-cured formulations when compared to those cured with the peroxide system (Figure 6). In the second stage, the active sulfurating agent substitutes the allylic hydrogens in rubber chains via a sulfur bridge, forming cross-link precursors. A primary vulcanizate network with a prevalence of polysulfidic cross-links is formed. In the third phase, the restructuration of the formed polysulfidic cross-links and modification of the rubber chains occur, and the final three-dimensional vulcanizate network is generated [27,51].

The experimental results showed that sulfur-cured rubber compounds based on NBR demonstrated a lower scorch time and lower optimum cure time when compared to corresponding compounds based on SBR. Simultaneously, both characteristics showed a decreasing trend with increasing content of the biopolymer, demonstrating its accelerating effect on the sulfur vulcanization of NBR-based formulations. However, the prolongation of the optimum cure time of SBR-based formulations with increasing content of lignosulfonate suggests that it has a retarding effect in the sulfur vulcanization of SBR.

Sulfur-cured SBR-based formulations demonstrated lower cross-link density, which seems to be a bit surprising as SBR contains a higher amount of butadiene structural units and thus a higher amount of active cross-linking sites. The higher cross-link density of composites based on NBR might be attributed to higher branching and entanglements of the rubber chains and polar structural units of NBR. Upon generation of chemical cross-links between adjacent entangled and branched rubber chains, the physical entanglements can also act as cross-linking points. Due to the presence of polar acrylonitrile units, stronger intermolecular interactions are formed between the chain segments, which could contribute to the higher cross-linking degree of NBR-based formulations, too. As also shown in Figure 4, the cross-link density of sulfur-cured composites based on NBR as well as SBR showed a slight decreasing trend with increasing content of calcium lignosulfonate. A possible explanation for this is that lignosulfonate could sterically hinder the formation of the cross-links between the chain segments. On the other hand, no negative influence of the biopolymer on the cross-link density of peroxide-cured composites suggest that organic peroxide in combination with a co-agent could cause the cross-linking of lignosulfonate or contribute to the formation of linkages between the biopolymer and the rubber matrix.

### 3.2. Physical–Mechanical Properties and Morphology

**Figure 8:**
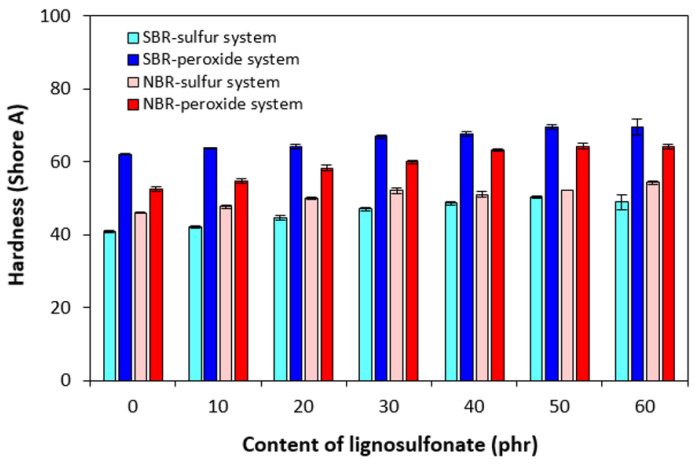
Influence of lignosulfonate content and curing system composition on hardness of composites.

The incorporation of lignosulfonate resulted in an increase of the composites’ hardness, suggesting that the hardness of the biopolymer was higher than that of the rubbers (Figure 8). The dependence of hardness on the type of rubber matrix and curing system composition was directly proportional to cross-link density (Figure 4). The highest cross-link density of composites based on SBR cured with the peroxide system was responsible for the highest hardness. On the other hand, the equivalent composites cured with the sulfur system with the lowest cross-linking degree exhibited the lowest hardness. The hardness of composites based on NBR ranged between the sulfur- and peroxide-cured composites based on SBR and was higher for peroxide-cured counterparts, again following the trend of cross-link density.

**Figure 9:**
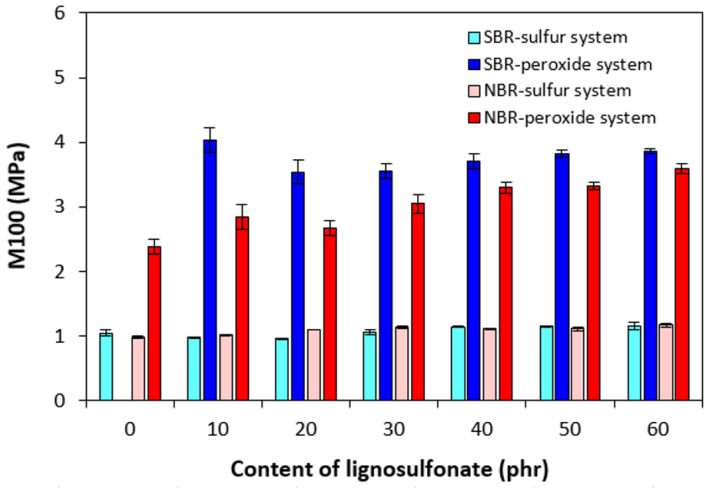
Influence of lignosulfonate content and curing system composition on modulus M100 of composites.

The higher cross-linking degree of both composite types cured with peroxide system was reflected in the higher modulus M100 (Figure 9). The M100 of NBR-based composites cured with the peroxide system was found to increase with increasing content of calcium lignosulfonate. The M100 of the peroxide-cured reference based on SBR was not possible to detect as this sample ruptured before reaching 100% elongation. The composites cured with the sulfur system demonstrated almost the same modulus with no influence on the biopolymer content. As also shown, the M100 of sulfur-cured composites was much lower than that of peroxide-cured equivalents due to their much lower cross-link density.

**Figure 10:**
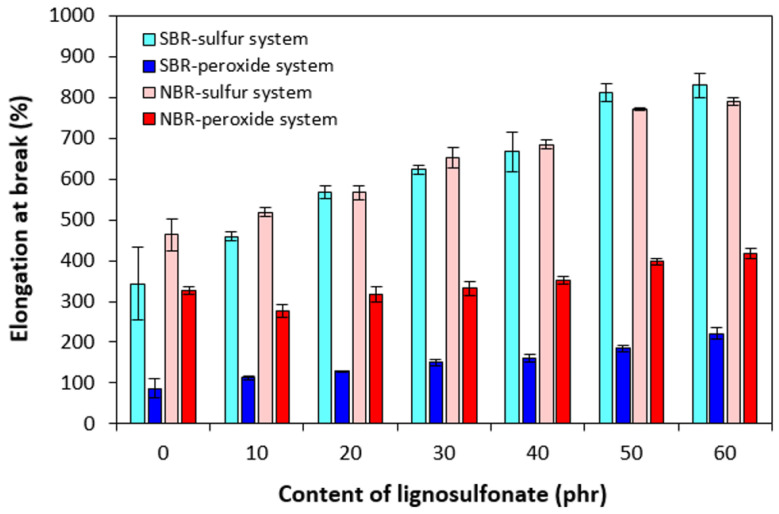
Influence of lignosulfonate content and curing system composition on elongation at break of composites.

On the other hand, the lower cross-link density of composites cured with the sulfur system resulted in their higher elongation at break (Figure 10). The elongation at break of the sulfur-cured composites based on NBR and SBR was very similar and was significantly dependent on the amount of the biopolymer. At the maximum lignosulfonate content, the elongation at break increased by less than 500% for SBR-based composites (from about 340% for the reference up to 830% for the composite with 60 phr of the biopolymer). In the case of sulfur-cured NBR-based composites, the elongation at break increased from 460% up to 790% by an increase in biopolymer content from 0 up to its maximum content. The higher the cross-link density, the higher the restriction of rubber chain segments’ elasticity and mobility, resulting in a decrease in elongation at break. The lowest elongation at break was exhibited by the SBR-based composites cured with the peroxide system with the highest cross-linking degree. As the cross-link density of peroxide-cured composites almost did not change with the change in lignosulfonate content, the elongation at break of the equivalent composites was much less dependent on lignosulfonate content. When compared to the references, the elongation at break increased by about 90% and 140% for the maximally filled composites based on NBR or SBR, respectively. Another factor contributing to the different elongation at break of sulfur- and peroxide-cured composites is the structure of the formed cross-links. Longer and more flexible sulfidic cross-links facilitate micro-Brownian motion of the chain segments between the cross-links, which results in better elastic properties in the sulfur cured rubber materials. Higher elasticity of the chain segments causes higher redistribution of the deformation strains within the rubber matrix, resulting in higher tensile characteristics, too. On the other hand, shorter and rigid carbon–carbon bonds restrict the orientation and mobility of the chain segments, which results in formation of local stress concentrations. To that correspond the worse dynamic and tensile behavior of peroxide-cured vulcanizates in general [27,29,52].

**Figure 11:**
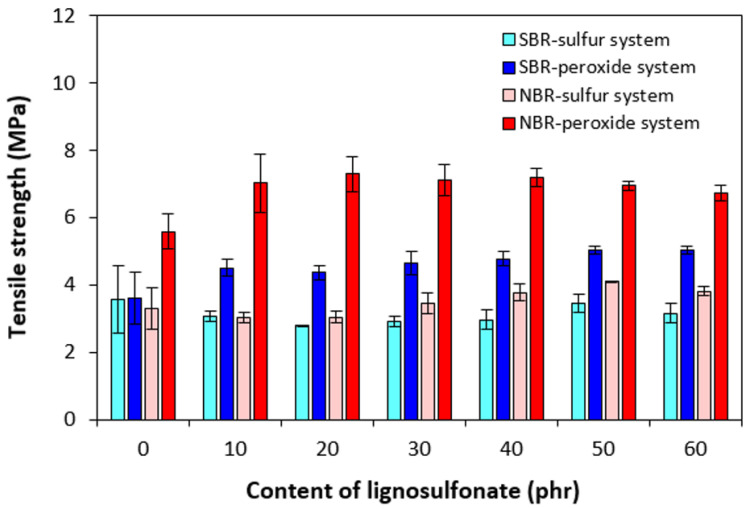
Influence of lignosulfonate content and curing system composition on tensile strength of composites.

From Figure 11, it becomes apparent that with the exception of the peroxide-cured reference sample based on NBR, the other reference samples exhibited roughly the same tensile strength. The tensile strength of sulfur-cured composites based on SBR was almost not influenced by the amount of the biopolymer, and it fluctuated only in a very low range of experimental values. The influence of lignosulfonate on the tensile strength of sulfur-cured NBR-based composites was also very low, though highly filled NBR-based composites exhibited slightly higher tensile strength when compared to equivalent sulfur-cured composites based on SBR. The application of the peroxide curing system resulted in the enhanced tensile behavior of composites, and the highest tensile strength was manifested by peroxide-cured composites based on NBR. By the incorporation of 10 phr of the biopolymer, an improvement in the tensile strength of both peroxide-cured composites was recorded when compared to the references. Then, no significant influence of lignosulfonate content on tensile strength was observed. The higher tensile characteristics of peroxide-cured vulcanizates can be attributed to the presence of a co-agent in peroxide vulcanization.

It has been reported that methacrylic acid zinc in the presence of organic peroxide can undergo in situ polymerization within the rubber matrix. Polymerized molecules of ZDMA can be physically adsorbed or chemically grafted onto rubber chains [53,54,55]. Thus, they form physical and chemical linkages in the rubber matrix. In addition, having polar-character ZDMA exhibits strong adhesion to polar materials [56,57]. Due to the presence of zinc ions, ZDMA can form ionic cross-links or ionic clusters, which can interact with polar functional groups of lignosulfonate. This leads to improvement in the adhesion and compatibility between the rubber and the biopolymer on the filler–rubber interface. Subsequent improvement in tensile strength was achieved. It becomes apparent from Figure 11 that higher tensile strength was demonstrated by composites based on NBR. As NBR, calcium lignosulfonate, and ZDMA are polar materials, their adhesion, compatibility, and formation of physical interactions between the components is higher when compared to non-polar SBR.

**Figure 12:**
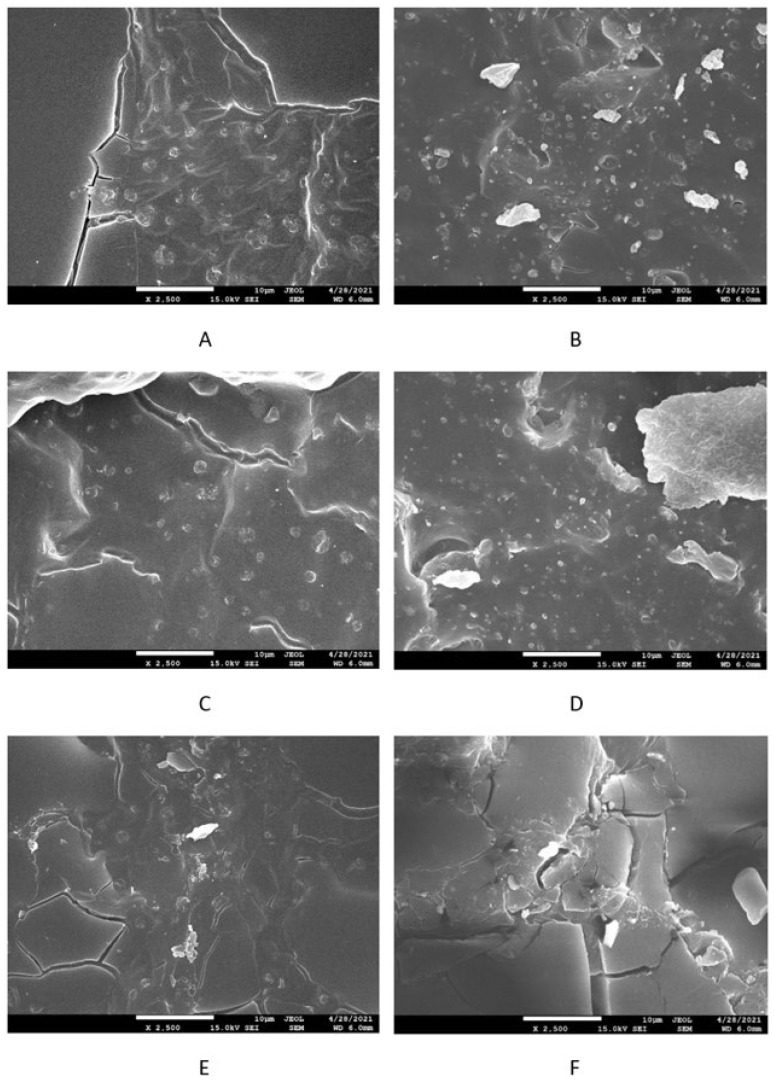
SEM images of sulfur-cured SBR-based composites filled with 10 phr of lignosulfonate (**A**), 30 phr of lignosulfnate (**C**), and 60 phr of lignosulfonate (**E**); sulfur-cured NBR-based composites filled with 10 phr of lignosulfnate (**B**), 30 phr of lignosulfnate (**D**), and 60 phr of lignosulfonate (**F**).

**Figure 13:**
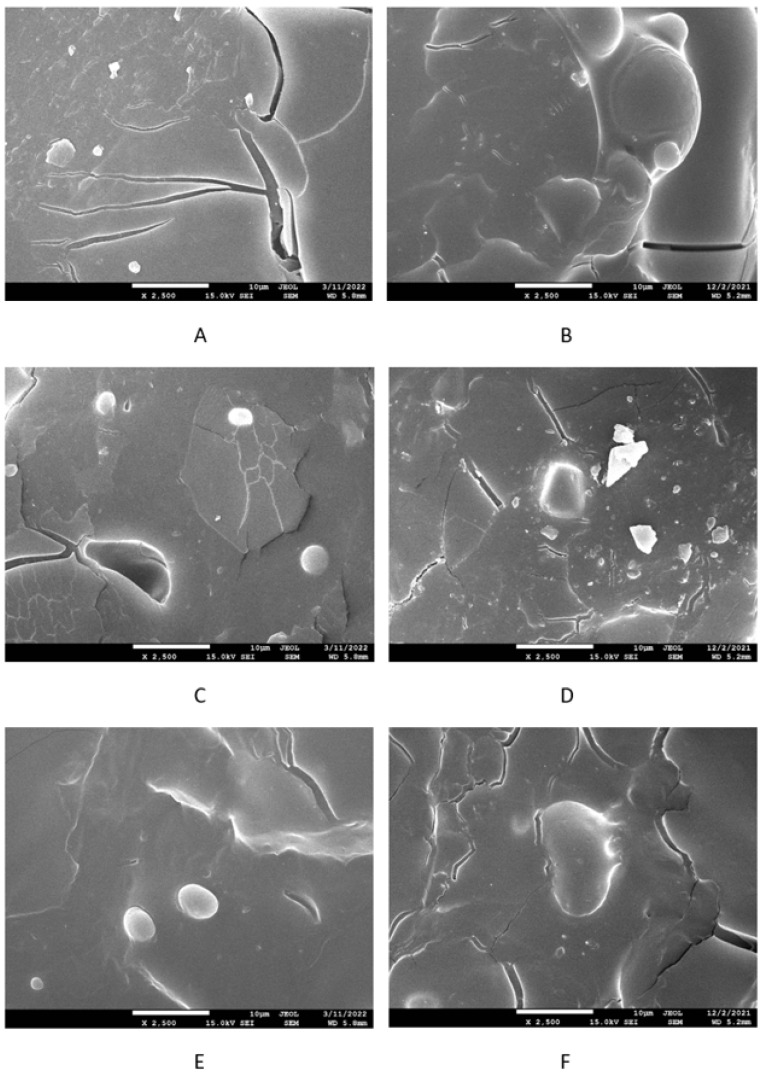
SEM images of peroxide-cured SBR-based composites filled with 10 phr of lignosulfonate (**A**), 30 phr of lignosulfnate (**C**), and 60 phr of lignosulfonate (**E**); peroxide cured NBR-based composites filled with 10 phr of lignosulfnate (**B**), 30 phr of lignosulfnate (**D**), and 60 phr of lignosulfonate (**F**).

The surface morphology of composites was studied by performing scanning electron microscopy (SEM). SEM images of the SBR-based composites cured with the sulfur system are presented in Figure 12 on the left side, while SEM images of the equivalent composites based on NBR are depicted in Figure 12 on the right side. Similarly, SEM images of peroxide-cured composites based on SBR and NBR are presented in Figure 13 on the left or right side, respectively. It is shown in Figure 12 that lignosulfonate tended to agglomerate in sulfur-cured composites. The agglomeration and worse dispersion of lignosulfonate within the rubber matrices cured with the sulfur system are the main reasons why lignosulfonate does not behave as a reinforcing filler. However, it should be remarked that although the application of lignosulfonate did not result in improvement in the tensile strength, no negative effect on the characteristic was recorded even at high lignosulfonate contents. Looking at Figure 13, one can see that the surface fractures of composites cured with peroxide system seem to be more compact, and better adhesion between the rubber and lignosulfonate on the interfacial region was achieved due to the presence of ZDMA. Some surface cracks visible on SEM images can be attributed to lignosulfonate, which was first melted during vulcanization at high temperatures. Then, the samples were frozen in liquid nitrogen, which might result in the brittleness of lignosulfonate. The cracks could then be caused by the fracturing of frozen samples for SEM analysis.

### 3.3. Dynamic–Mechanical Analysis

**Figure 14:**
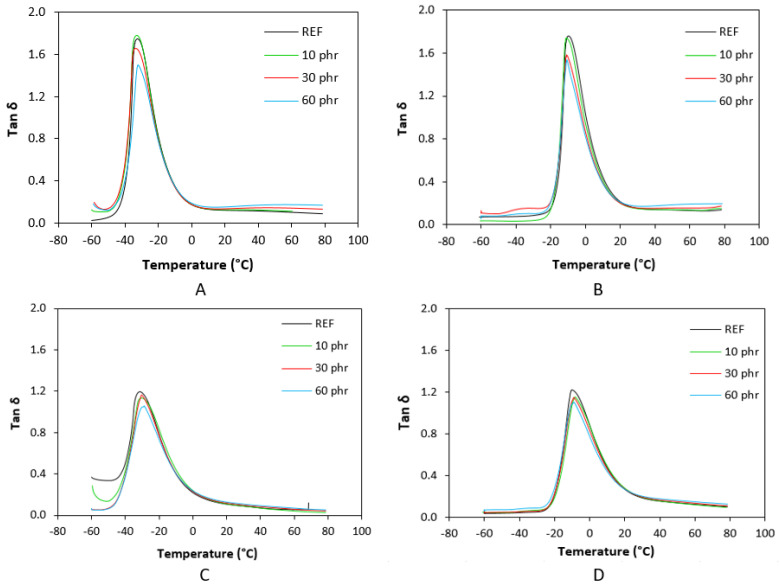
Temperature dependences of loss factor tan *δ* for SBR-based composites cured with sulfur system (**A**), NBR-based composites cured with sulfur system (**B**), SBR-based composites cured with peroxide system (**C**), and NBR-based composites cured with peroxide system (**D**).

The temperature dependences of loss factor tan *δ* for tested materials are illustrated in Figure 14, while the values of tan *δ* at chosen temperatures are summarized in Table 1, Table 2, Table 3 and Table 4. Tan *δ* is a ratio of loss and storage modulus and corresponds to the energy lost to energy absorbed and returned by the system pre unit cycle. With the rise in temperature, tan *δ* reaches the maximum in the transition region, followed by a subsequent decrease in the rubbery region. The peak maximum corresponds to the glass transition temperature *Tg*. In glassy region below *Tg*, the damping is low, as the chain segments are immobilized and deformations are mostly elastic due to limited propensity of the chain segments to viscous flow. In the rubbery region, damping is low, because the rotational movement of the chain segments enables free movement with minimum resistance to flow. In the transition region, damping and energy dissipation is high due to the inception of micro-Brownian movement in the chains and chain stress relaxation, although not all the segments are able to participate in such relaxations together. As shown in Figure 14, the application of lignosulfonate resulted in the lowering of the tan *δ* peak and a reduction in area in the transition region, resulting in lower energy dissipation, which can be attributed to a higher storage modulus in this region. In all cases, the lowest area in a transition region was demonstrated by composites filled with maximum lignosulfonate content.

From Figure 14 and Table 1, Table 2, Table 3 and Table 4, it becomes apparent that sulfur- as well as peroxide-cured composites based on SBR exhibited a lower *Tg* when compared to the corresponding composites based on NBR. It is a logical reflection of the higher amount of rubbery butadiene units in SBR’s structure. The *Tg* of sulfur-cured composites based on SBR moved around −33 °C, while the *Tg* of peroxide cured composites was slightly higher (around −31 °C). This can be attributed to the higher cross-linking degree of peroxide-cured composites, suggesting that increased cross-link density restricts the chain segments’ elasticity and mobility, thus elevating *Tg*. The same can be applied to composites based on NBR. The higher cross-link density of peroxide cured composites resulted in an increase in *Tg* by about 2 °C (from about −11 °C for sulfur-cured composites to around −9 °C for peroxide-cured equivalents).

It can also be seen from Table 1, Table 2, Table 3 and Table 4 that the *Tg* in some samples increases, while in others, it slightly decreases with increasing filler content. However, there was no recorded direct influence of the filler on *Tg* regarding the type of rubber matrix or curing system composition. It can also be stated that the values tan *δ* in the glassy and rubbery region were significantly influenced neither by the amount of lignosulfonate nor by the type of curing system applied. This may suggest a lack of strong filler–rubber interactions, implying no molecular-level changes.

## 4. Conclusions

Calcium lignosulfonate-filled formulations based on SBR and NBR were cured with sulfur as well as peroxide curing systems. The results revealed that peroxide-cured composites exhibited a lower scorch time, higher torque difference, and higher cross-link density. Higher cross-link density resulted in a higher hardness and modulus and a lower elongation at break in the corresponding composites. Peroxide-cured composites demonstrated higher tensile strength, which can be attributed to higher compatibility and adhesion and between the filler and the rubber on their interface due to the presence of methacrylic acid zinc salt in peroxide vulcanization. The highest tensile strength of composites based on NBR can be attributed to the polar nature of the rubber, the biopolymer, and the co-agent resulting in the best adhesion and physical interactions between the components. The application of lignosulfonate in all rubber systems resulted in an increase in elongation at break and hardness, while a more significant influence on elongation at break was recorded for sulfur-cured counterparts. The presence of the biopolymer in the compounds did not significantly influence the *Tg* of the composites and tan *δ* in the glassy and rubbery region. The decrease in the tan *δ* peak and lowering of the area in the transition region with the increase in lignosulfonate content point to reduced damping in that region.

## Figures and Tables

**Table 1:** Glass transition temperature *Tg* and loss factor tan *δ* of SBR-based composites cured with sulfur system.

Lignosulfonate (phr)	*Tg* (°C)	tan *δ * at *Tg*	tan *δ * (−50 °C)	tan *δ * (−20 °C)	tan *δ * (0 °C)	tan *δ * (20 °C)	tan *δ * (50 °C)
0	−32.4	1.74	0.06	0.83	0.18	0.12	0.11
10	−33.0	1.77	0.11	0.81	0.17	0.13	0.12
30	−33.6	1.66	0.14	0.79	0.18	0.14	0.15
60	−32.4	1.50	0.12	0.77	0.18	0.15	0.17

**Table 2:** Glass transition temperature *Tg* and loss factor tan *δ* of SBR-based composites cured with peroxide system.

Lignosulfonate (phr)	*Tg* (°C)	tan *δ * at *Tg*	tan *δ * (−50 °C)	tan *δ * (−20 °C)	tan *δ * (0 °C)	tan *δ * (20 °C)	tan *δ * (50 °C)
0	−31.3	1.20	0.33	0.78	0.22	0.10	0.06
10	−30.7	1.13	0.14	0.82	0.24	0.11	0.05
30	−30.4	1.16	0.06	0.77	0.23	0.12	0.07
60	−28.9	1.05	0.06	0.73	0.23	0.13	0.08

**Table 3:** Glass transition temperature *Tg* and loss factor tan *δ* of NBR-based composites cured with sulfur system.

Lignosulfonate (phr)	*Tg* (°C)	tan *δ * at *Tg*	tan *δ * (−50 °C)	tan *δ * (−20 °C)	tan *δ * (0 °C)	tan *δ * (20 °C)	tan *δ * (50 °C)
0	−9.8	1.76	0.07	0.15	1.03	0.21	0.13
10	−10.9	1.74	0.04	0.14	0.96	0.20	0.14
30	−10.7	1.58	0.10	0.21	0.88	0.21	0.16
60	−10.7	1.53	0.08	0.21	0.82	0.21	0.19

**Table 4:** Glass transition temperature *Tg* and loss factor tan *δ* of NBR-based composites cured with peroxide system.

Lignosulfonate (phr)	*Tg* (°C)	tan *δ * at *Tg*	tan *δ * (−50 °C)	tan *δ * (−20 °C)	tan *δ * (0 °C)	tan *δ * (20 °C)	tan *δ * (50 °C)
0	−10.1	1.22	0.04	0.22	0.89	0.27	0.14
10	−8.2	1.15	0.05	0.18	0.88	0.28	0.14
30	−8.9	1.15	0.05	0.23	0.83	0.27	0.15
60	−8.9	1.10	0.07	0.28	0.77	0.27	0.16

## Data Availability

The original contributions presented in this study are included in the article. Further inquiries can be directed to the corresponding author.
